# Tiered dietary exposure assessment of steviol glycosides in the Belgian population

**DOI:** 10.1017/jns.2023.13

**Published:** 2023-02-21

**Authors:** Joris Van Loco, Raphael Janssens, Séverine Goscinny, Els Van Hoeck, Christiane Vleminckx, Mirjana Andjelkovic

**Affiliations:** 1 Chemical and Physical Health Risks, Sciensano, J. Wytsmanstraat 14, Brussels 1050, Belgium; 2 Clinical and Experimental Endocrinology, KU Leuven, Herestraat 49, Leuven 3000, Belgium; 3 Risk and Health Impact Assessment, Sciensano, J. Wytsmanstraat 14, Brussels 1050, Belgium; 4 Organic Contaminants and Additives, Sciensano, J. Wytsmanstraat 14, Brussels 1050, Belgium

**Keywords:** Dietary intake, Exposure, Food additive, Steviol glycosides, Sweeteners

## Abstract

The objective of the present study was to assess the dietary intake of steviol glycosides in the Belgian population and to conduct a risk assessment by comparing the estimated intakes to the acceptable daily intake (ADI). A tiered approach was adopted in this study. First, a Tier 2 assessment was performed using maximum permitted levels. Next, the calculations were refined because market share data were used (refined Tier 2). Finally, the actual concentration data of 198 samples purchased from the Belgian market were used for Tier 3 exposure assessment. Based on a Tier 2 assessment, the ADI was exceeded for the high-consumer children population. However, the results of a more refined exposure assessment (Tier 3) of high consumers (P95) within the child, adolescent and adult populations were 13·75, 10 and 6·25 % of the ADI, respectively, using mean analytical results. Even with more conservative refined approaches, the estimated daily intake remained below 20 % of ADI. Flavoured drinks, flavoured fermented milk products and jams, jellies, and marmalades were the top three contributing food groups to steviol intake at 26–49 %, 12–27 % and 5–13 %, respectively. Despite the high concentrations (up to 94 000 mg/kg) of steviol glycosides in tabletop sweeteners, their contribution to the total intake remains low. The impact of the use of food supplements on the total intake was also considered to be limited. It was concluded that there was no risk for the Belgian population related to dietary exposure to steviol glycoside.

## Graphical abstract

**Figure:**
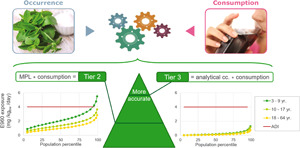

## Introduction

Growing awareness of the health implications of high-calorie diets has driven the need to reduce sugar intake^(1–5)^. Overweight and obesity have reached epidemic proportions in the European Region, affecting 59 % of the adults. Approximately 30 % of the school-aged children and one in four adolescents are overweighed or obese^(6)^. The frequent consumption of sugared drinks has been associated with overweight and obesity, a higher risk of cardiovascular diseases, diabetes mellitus, some cancers and mental health problems^(6,7)^. Policy makers are taking initiatives to limit sugar intake with prevention and legislative initiatives such as sugar tax and front-of-pack labelling^(8–11)^. The WHO recommend to limit free sugars intake to less than 10 % of total energy intake^(12)^. Consequently, sugar is replaced with low-/no-calorie sweeteners. Different independent studies report a rise in sweetener consumption in the past several years^(13–17)^. The market share of natural sweeteners, such as steviol glycosides, is forecasted to expand faster than that of other (synthetic) sweeteners^(13,17)^.

Over the past few decades, several sugar substitutes have been developed and authorised as food additives by the European Commission (Commission Regulation N° 1333/2008)^(18)^. Examples include aspartame (E 951), saccharin (E 954), sucralose (E 955), thaumatin (E 957), steviol glycosides (E 960a-c) and neotame (E 961). Steviol glycosides are authorised in different food categories at different maximum levels, except for quantum satis (QS) levels in tabletop sweeteners^(19)^. Steviol glycosides are extracted from the leaves of the stevia plants or prepared via enzymatic production from stevioside^(20,21)^. This sweetener is 300 times the sweetness of sugar but has an almost negligible effect on blood glucose levels; hence, it is considered an attractive substitute for sugar^(22)^. In Europe, the European Food Safety Authority (EFSA) established in 2010 an acceptable daily intake (ADI) of 4 mg/kg bw/d for steviol glycosides, expressed as steviol equivalents^(23)^.

Studies on dietary exposure to steviol glycosides in Europe are limited^(24–27)^. A global review of dietary intake of low-calorie sweeteners was recently conducted by Martyn *et al.*^(28)^. Although the consumption of steviol glycosides can be considered safe (below the ADI) in Europe, high consumption scenarios with children have indicated potential exceedance of the ADI^(26,27,29)^. The authors mentioned that these scenarios were very conservative, and that the intake of steviol glycosides was not a concern. Similar observations were made by the Food Standards Australia New Zealand^(30)^. In Belgium, no specific steviol glycoside dietary exposure study has been conducted in the general population. De Winter *et al.* studied the intake of steviol glycosides in children with type 1 diabetes mellitus in Belgium. They concluded that in Tier 2 (using reference concentration data) and Tier 3 (using measured concentration data) exposure assessments, the exposure is above the ADI for young children in the most conservative scenario^(27)^. The present study aimed to perform a tiered and refined dietary exposure assessment of the Belgian general population (aged 3–64 years old) to steviol glycosides and a subsequent risk assessment.

## Methods

### Data collection

#### Steviol occurrence data

A foodstuff search for ‘E 960’ and ‘steviol glycosides’ in food and drinks was performed in the Global New Products Database (GNPD) from Mintel to establish a shopping list, complemented with local information from supermarket visits (local label survey). A sampling campaign was performed in Belgium between October 2019 and February 2020 in major supermarkets (Colruyt, Carrefour, Delhaize, Aldi, Lidl and Albert Hein), pharmacies and para-pharmacies (e.g. Di). Additionally, shopping at ‘RegimeProteine.be’ and Holland & Barrett was done online for specific (protein bars and tabletop sweeteners) or house-brand products. Storage conditions were as required by the type of product (e.g. freezer for frozen foods, refrigerated for fresh products like yoghurts), and expiration dates were closely monitored.

#### Food consumption data

The exposure assessments were performed based on whole food consumption data from the Belgian Food Consumption Survey conducted in 2014 (BNFCS2014)^(31)^. More details on this survey can be found in previously published studies^(31,32)^. Only participants with two completed 24 h recalls were included in the exposure assessment. Before performing the dietary exposure assessment, consumption records which were codified according to the BNFCS2014 classification and FoodEx2 classification system were linked to the food categorisation system (FCS), as described in Annex II of Regulation (EC) No. 1333/2008^(18,33)^.

### Chemical analysis

Steviol glycosides were analysed using a previously reported in-house validated method^(25)^. Briefly, the procedure included sample extraction, followed by acid hydrolysis under heating conditions to transform the steviol glycosides into isosteviol prior to measurements using liquid chromatography in combination with tandem mass spectrometry (LC-MS/MS). This methodology allows direct quantification of the concentration of steviol glycosides as steviol equivalents. The extraction step was optimised for steviol glycosides in all matrices. In contrast to beverages, solid matrices must be subjected to extraction. Steviol glycosides are mid-polar compounds that can be extracted using polar solvents, such as water and methanol. However, to accommodate different types of food products, mixtures of solvents are used to isolate steviol glycosides from matrix substances, such as acidified water for protein precipitation, dichloromethane (DCM) for fat removal or gelatine denaturation. Table 1 provides an overview of the extraction protocols for different solid matrices. Once steviol glycosides were extracted, acid hydrolysis transformed them into isosteviols. The hydrolysis mixture comprised 100 μL of the extract and 940 μL of a 2·5 % sulphuric acid (H_2_SO_4_) solution. The reaction was carried out for 15 h at 80 °C. After acid hydrolysis, the mixture was extracted twice with 800 μL methyl tert-butyl ether. The organic phases were pooled and dried under a gentle stream of nitrogen. The residue was dissolved in 5 mL of an acetonitrile–water mixture (7:3, v/v) prior to LC-MS analysis.

**Table 1.** Extraction solutions for the solid food matrices

Matrix	Sample take (g)	Extraction solution
Sweets	0·25	5 mL of methanol (MeOH): water with 2·5 % sulphuric acid (H_2_SO_4_) (1:1)
Breakfast cereals and cereal-based food	2	10 mL MeOH: water with 2·5 % H_2_SO_4_ (1:1)
Other solid foods (e.g. dairies, deserts and chocolate confectionaries)	1	2 mL water with 2·5 % H_2_SO_4_
Sauces	0·5	5 mL water with 2·5 % H_2_SO_4_
Tabletop sweeteners and dry food supplements	1	50 mL water
Jams	1	2 mL water with 2·5 % H_2_SO_4_

Next, the extracts were injected in the Acquity UPLC® system (sample and quaternary solvent manager, column oven) hyphenated to a Xevo™ TQ-S triple quadrupole mass spectrometer both from Waters (Milford, MA, USA), equipped with an Acquity UPLC® HSS C18 column (dimensions: 100 × 2·1 mm and particle size: 1·7 μm) at 45 °C. Isosteviol was eluted for 4·2 min using mobile phase (A) 0·1 % formic acid and (B) acetonitrile with 0·1 % formic acid for 6 min with a linear gradient from 25 to 95 % B for 4 min, followed by isocratic flow for 0·5 min and back to initial conditions (25 % B) for 0·5 min and kept for 1 min. The flow rate used was 0·40 mL/min, and the injection volume was 5 μL.

Electrospray ionisation (ESI) was applied in the negative mode. Following conditions for MS parameters were utilised: capillary voltage, 2·0 kV; cone voltage, 30 V; source temperature, 150 °C; desolvation temperature, 500 °C; nitrogen used as a cone and desolvation gas with flow rates of 150 and 1000 L/h, respectively, and argon as collision gas with a flow rate of 0·17 mL/min. The multiple reaction monitoring (MRM) transitions, as well as the cone voltages and collision energies, were optimised to MRM=317·20 Da >317·20 Da with 10·0 eV collision energy and MRM=385·20 Da >317·20 Da with 30·0 eV collision energy. Instrument control, data acquisition and data analysis were performed using Masslynx™ software (version 4.1., Waters). During data analysis, all chromatograms were processed using TargetLynx™ software (Waters), and quantification was performed using matrix-matched calibration curves with concentrations ranging from 5 to 100 ng/mL. The extracts were diluted if necessary. The quality control sample was recovered by fortifying the blank extract with rebaudioside A at 100 ng/mL steviol equivalent. After acid hydrolysis, the recovery was used to correct the concentrations measured for the same batch only when the yield was below 75 %.

### Dietary exposure assessment

Exposure assessment was performed following a tiered approach, as recommended by the EFSA^(34)^. The first tier, which utilises the household budget survey method focusing on household expenditures on goods and services, was not explored in this study^(35)^. The Tier 2 and Tier 3 approaches combined food consumption data with the maximum permitted level (MPL) or actual concentration of the additive in each food category, respectively. In the Tier 2 approach, the assessment was carried out by multiplying the MPLs of the steviol glycoside additive with the refined consumption data for the different food groups of BNFCS2014^(31)^. Individual intake of the additive was estimated using the following equation:
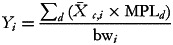
where *Y_i_* is the daily additive intake of a given individual *i* (mg/kg body weight/d), bw*_i_* is the self-reported body weight of a given individual, 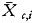 is the average amount of a commodity consumed per day (kg) and MPL*_d_* is the maximum amount allowed by Regulation (EC) No1333/2008 for the studied additive in the foodstuff (mg/kg_food_)^(36)^.

Four exposure scenarios were developed: (a) Tier 2 including only MPLs (T2), (b) Tier 2 with an assumed usage level of 12 000 mg_steviol eq_/kg for the food category ‘tabletop sweeteners’ which are authorised as quantum satis (T2QS). The assumed usage level was obtained from EFSA evaluation of steviol glycosides^(37)^. These two scenarios were further refined by only including food categories in which steviol glucosides are used as a food additive and are present in the Belgian market: (c) refined Tier 2 including only legislative MPLs of products on the Belgian market (rT2) and (d) refined Tier 2 with an assumed usage level of 12 000 mg_steviol eq_/kg for the food category ‘tabletop sweeteners’ (rT2QS).

In the Tier 3 approach, MPL was replaced by the actual level of steviol glycosides in food. Three exposure scenarios were assessed: mean analytical concentrations per food group (T3), maximum analytical concentrations per food group (T3max) and maximum concentrations including food supplements (T3maxfs). The latter was motivated by the fact that dietary supplements are episodically consumed. The consumption frequencies were adjusted for the time of year, and the posology of food supplements was based on their intended use: for children (<10 years) or for adults (≥10 years).

The Tier 3 estimated exposure was modelled using a 2-part model, whereas the Tier 3 refined scenarios were modelled using the 3-part model available in Statistical Program to Assess Dietary Exposure (SPADE)^(38)^. This method eliminates intra-individual variance and transforms data into normally distributed data. The usual intake distribution was weighted and adjusted for the age and sex distribution of the Belgian population, and for the day of the week and season. An overview of the different scenarios is given in Table 2.

**Table 2.** Overview of the tiered exposure scenarios with selected parameters

Exposure scenario	Code	Food category	Steviol glucosides use level	Tabletop sweeteners
Tier 2	T2	All	MPL	
	T2QS	All	MPL	12 000 mg/kg
Refined Tier 2	rT2	BE^a^	MPL	
	rT2QS	BE	MPL	12 000 mg/kg
Tier 3	T3	BE	Mean concentration^b^	Mean concentration
	T3max	BE	Maximum concentration^c^	Maximum concentration
	T3maxfs	BE	Maximum concentration + food supplement posology	Maximum concentration

a BE: only food categories in which products containing steviol glycosides were found within the Belgian market.

b Mean concentration per food (sub)category (Supplementary Table 1).

c Maximum concentration per food (sub)category (Supplementary Table 1).

The effects of uncertainties in the exposure estimation were qualitatively assessed. Possible sources of variability or imprecision in the exposure estimates are discussed. The impact was estimated based on expert knowledge as overestimation and/or underestimation, assigning different levels (large, medium and small).

## Results

### Steviol glycoside concentration levels

In total, 198 samples were purchased from Belgium supermarkets. Based on a local label survey and Mintel data, the sample size represented the entire Belgian market. The contents of the samples were analysed (Supplementary Table 1). The results vary according to the authorised levels but also within the food groups, depending on the type of food product and the food formulation, as steviol glycosides are rarely used as the sole sweetener substance. All samples were in compliance with the MPLs. For most of the samples (88 %), sugar and polyols are often used in conjunction with steviol glycosides and, to a lesser extent, other intense sweeteners (e.g. acesulfam-k and sucralose). The highest concentration was found in tabletop sweeteners, specifically in tablet form.

### Dietary intake

With increasing precision, a tiered dietary intake was assessed using seven exposure scenarios. There were two scenarios in Tier 2 (T2 and T2QS) and two refined Tier 2 scenarios (rT2 and rT2QS). The ‘QS’ indicates intake from the food category ‘tabletop sweeteners’, which are authorised as quantum satis (QS). In the Tier 2 scenarios, all authorised food groups for steviol glycosides were considered, and both Tier 2 scenarios were further refined by including only food categories present in the Belgian market. The results for the Tier 2 and refined Tier 2 scenarios are presented in Table 3.

**Table 3.** Tier 2 estimated exposure to steviol glycosides (mg_stevio eq._/kg bw/d) in the Belgian population* using the maximum permitted levels for the four exposure scenarios: Tier 2 (T2 and T2QS) and refined Tier 2 (rT2 and rT2QS)

Exposure scenario	Population age groups	*N*	Estimated exposure (mg_stevio eq._/kg bw/d)		
			Mean	P50	P95
T2	Children (3–9 years)	1948	2·25	2·07	4·23
	Adolescents (10–17 years)	1825	1·48	1·36	2·76
	Adults (18–64 years)	2392	1·07	0·97	2·09
T2QS	Children (3–9 years)	1948	2·24	2·06	4·23
	Adolescents (10–17 years)	1825	1·50	1·38	2·81
	Adults (18–64 years)	2392	1·09	0·99	2·15
rT2	Children (3–9 years)	1934	1·86	1·74	3·38
	Adolescents (10–17 years)	1813	1·30	1·20	2·46
	Adults (18–64 years)	2331	0·79	0·69	1·69
rT2QS	Children (3–9 years)	1934	1·85	1·73	3·39
	Adolescents (10–17 years)	1813	1·31	1·22	2·50
	Adults (18–64 years)	2336	0·81	0·72	1·74

The mean estimated exposures to steviol glucosides remained below the ADI for all age classes in the two Tier 2 scenarios with intake levels for steviol glycosides at 1·1–2·2 mg/kg bw/d. In comparison with the ADI of 4 mg/kg bw/d, the mean exposure in the T2QS scenario was 27, 38 and 56 % of the ADI for adults, adolescents and children, respectively. Additionally, in two scenarios (T2 and T2QS), the results imply that high consumers (P95) among children may be exposed to a level higher than the ADI (106 % of the ADI). As expected from the refinement approach, the exposure of all populations were lower in the refined exposure scenarios (rT2 and rT2QS) with mean intake levels 0·8–1·9 mg/kg bw/d or 20–47 % of the ADI. No exceedance of the ADI was observed in the refined Tier 2 scenarios with the highest intake for children (P95) at 85 % of the ADI. The results of all Tier 2 and refined Tier 2 scenarios indicate that children were more exposed to steviol glycosides than adolescents and adults because of their lower body weights.

The refined Tier 2 scenario was further refined using the actual concentration data for the Tier 3 mean (T3) and the maximum analytical concentration scenario (T3max). The latter scenario was complemented by the intake of food supplements (T3maxfs) (Table 4). The Tier 3 scenario results indicated that the child population was more exposed to steviol glycosides than the adolescent or adult population, similar to the Tier 2 and refined Tier 2 results. The exposure of high consumers (P95) within the child, adolescent and adult populations were 13·75, 10 and 6·25 % of the ADI, respectively, using mean analytical results (T3). The estimated exposure of the entire population remained below 20 % of the ADI with the mean estimated exposure below 7 % of the ADI in all Tier 3 scenarios.

**Table 4.** Tier 3 estimated exposure to steviol glycosides (mg_stevio eq._/kg bw/d) in the Belgian population using actual concentration data for the Tier 3 mean (T3) and maximum (T3max) analytical concentration scenario including food supplements posology (T3maxfs)

Exposure scenario	Population age groups	*N*	Estimated exposure (mg_stevio eq._/kg bw/d)		
			Mean	P50	P95
T3	Children (3–9 years)	1308	0·20	0·15	0·55
	Adolescents (10–17 years)	1216	0·14	0·10	0·40
	Adults (18–64 years)	1495	0·08	0·05	0·25
T3max	Children (3–9 years)	1308	0·26	0·18	0·78
	Adolescents (10–17 years)	1216	0·23	0·15	0·70
	Adults (18–64 years)	1495	0·13	0·07	0·42
T3maxfs	Children (3–9 years)	1308	0·27	0·18	0·80
	Adolescents (10–17 years)	1216	0·24	0·16	0·71
	Adults (18–64 years)	1495	0·15	0·08	0·48

Food categories contributing to exposure to steviol glycosides were analysed, and the contribution of each food category to the Tier 3 mean analytical concentration scenario (T3) is presented in Table 5. The categories ‘*Flavoured drinks*’, ‘*flavoured fermented milk products*’ and ‘*jam, jellies and marmalades*’ were the top three contributing food groups to steviol glycosides intake at 26–49 %, 12–28 % and 5–13 %, respectively. The intake for all other food categories was below 10 %.

**Table 5.** Food categories contributing to exposure to steviol glycosides (E 960) for the Tier 3 mean analytical concentration scenario (T3)

FCS category number	FCS food category	Children 3–9 years (*N* 1308)	Adolescents 10–17 years (*N* 1216)	Adults 18–64 years (*N* 1495)
01.4	Flavoured fermented milk products including heat-treated products	22·2 %	12·4 %	27·6 %
03	Edible ices	3·1 %	5·9 %	4·3 %
04.2.5.2	Jam, jellies and marmalades and sweetened chestnut purée as defined by Directive 2001/113/EC	6·9 %	4·7 %	12·9 %
05.1	Cocoa and chocolate products as covered by Directive 2000/36/EC	4·7 %	4·0 %	8·0 %
05.2	Other confectionery including breath-freshening micro sweets	8·7 %	3·6 %	2·1 %
06.3	Breakfast cereals	0·0 %	0·6 %	2·4 %
07.2	Fine bakery wares	1·9 %	1·7 %	0.8 %
11.4	Tabletop sweeteners	0·0 %	0·0 %	1·8 %
12.6	Sauces	8·8 %	9·2 %	3·9 %
13.3	Dietary foods for weight control diets	0·0 %	0·8 %	1·0 %
14.1.4	Flavoured drinks	36·9 %	48·9 %	26·4 %
14.2.1	Beer and malt beverages	0·0 %	0·7 %	0·5 %
15.1	Potato-, cereal-, flour- or starch-based snacks	4·5 %	5·7 %	5·2 %
17.1	Food supplements supplied in a solid form	2·2 %	1·8 %	3·2 %
17.2	Food supplements supplied in a liquid form	0 %	0 %	0 %
	Total	100 %	100 %	100 %

## Discussion

### Tier 2 approach

Food additives authorised as QS are generally excluded from Tier 2 intake assessment. However, in the latest EFSA evaluation of steviol glycosides, a maximum level of 12 000 mg/kg has been used^(29,37)^. The difference between scenarios (r)T2 and (r)T2QS is that (r)T2 excludes the ‘tabletop sweetener’ food category. However, a significant difference could not be observed in the estimated exposures between the scenarios due to the limited contribution of ‘tabletop sweeteners’ (Table 3) to the total intake, despite the associated high maximum use level. The relatively small frequency of tabletop sweetener consumption by in particular adolescents (1·2 %) and adults (20·7 %) could explain the small contribution of this food category. Notably, a potential underestimation could be related to the assumption of 12 000 mg/kg, as retrieved from the EFSA opinion^(39)^. This level is in line with the maximal concentration measured in samples labelled as ‘steviol glycosides sweetener in powder form’ typically used to sweeten hot beverages. However, the significantly higher levels (max. 94 000 mg/kg) were detected in samples labelled as ‘sweeteners in tablet form’ and used to sweeten hot beverages (Supplementary Table 1). Therefore, we recommend using a higher value as the maximal reported use level in future (refined) Tier 2 exposure assessments because of the widespread use of tablet forms of sweeteners.

Further refinement of the Tier 2 intake scenarios by excluding food categories with steviol glycosides not present in the Belgian market (rT2 and rT2QS) did not exceed ADI (Table 3). This can be explained by the fact that no products with steviol glycosides were found in the Belgian market for approximately one-third of the authorised food categories. It also needs to be stressed that Tier 2 exposure estimates are conservative due to the use of MPLs, but also when linking FCS categories with consumption data. Food categories across various food classification systems are not directly comparable, and a link between food classification systems is needed to assign MPLs to the correct food group in the BNFCS2014. As demonstrated in similar studies on dietary intake of sweeteners, refined intake estimates significantly impacted the overall conclusions^(24,25)^. The use of databases such as the GNPD or local label surveys is strongly recommended because they can help refine the results of food additive intake assessments^(40)^.

In their 2015 opinion, EFSA evaluated the exposure to steviol glycosides from its use as a food additive using MPLs and the extension of its use at the levels proposed by the applicant^(29)^. The results of the EFSA exposure assessment performed in 2015 indicate that the mean estimates remained below the ADI for all population groups, with children being the most exposed group. However, the exposure levels in the EFSA 2015 evaluation for Belgium were lower than the Tier 2 (T2 and T2QS) estimates in the present study. This can be explained by (i) the food categories not authorised in 2015, such as mustard and food supplements in chewable form; (ii) the use of older consumption studies in the evaluation of EFSA (2008 Regional Flanders (toddlers and children) and 2004 Belgian Food Consumption^(32)^ (adolescents and adults); and (iii) the exclusion of some food categories from the assessment. Here, the impact of the fine bakery wares was important because the MPL is restricted to ‘only essoblaten-wafer paper’. Applying MPL to the whole food group of fine bakery wares in the present study led to a conservative overestimation. This conservative overestimation of this study is also visible when comparing the results with Tier 2 intake results from studies in Italian and Irish populations^(24,25)^.

### Tier 3 approach

When comparing the scenarios, higher values were obtained for the maximum analytical concentration scenarios than for the mean analytical scenario, which is explained by the definition of the scenarios. The highest Tier 3 exposure was obtained for the T3maxfs scenario because of the inclusion of food supplement intake assessment. This scenario can be considered a worst-case Tier 3 scenario because of the maximum use levels and the assumed intake of food supplements. It is generally expected that food supplements are only consumed sporadically or during a limited period of the year. It was also observed that food supplements made a small contribution to total estimated exposure. This is in contrast to the results of Buffini *et al.*, where almost 90 % of the steviol exposure was allocated to solid food supplements^(25)^. These results with food supplements are important findings of this study, since few studies have used food supplements in the exposure assessment for additives.

Brand loyalty has not been specifically investigated. However, in the T3max scenario, all food categories were set to the maximum analytical concentration, which is more conservative than in the brand-loyal scenario. As expected, an increase in the total intake was observed compared to the T3 mean analytical concentration scenario, but no impact on the risk evaluation outcome was observed (Table 4). Since both the T3max and T3maxfs scenarios overestimated the intake for the general Belgian population, it can be concluded that exposure to steviol glycosides is far below the ADI for all studied age groups. Consequently, health effects from excess steviol glycoside exposure at current use levels are unlikely.

The present study refines the findings of De Winter *et al.* concerning the exposure of children in Belgium to steviol glycosides^(27)^. However, the present findings suggest that exceedance of the ADI for the child population is very unlikely because the exposure of the high (P95) and very high consumer group (P99) in the T3max scenario to steviol glycosides was 0·78 and 1·28 μg/kg bw/d.

Further research should aim to provide more data on the levels of steviol glycosides in foodstuffs targeting specific population groups, such as people in weight restriction programs or following specific diets such as diabetic and PKU patients. There is also a need to follow new marketing trends, new consumption patterns and the effects of (governmental) initiatives on sugar reduction and their potential effects on sweetener intake.

### Identification of the major contributors

The contribution of flavoured drinks and flavoured fermented milk products to the total intake (Table 5) is in line with the conclusions of EFSA and other studies on sweeteners and steviol glycosides^(24–26,41)^. It should be noted that the category contributions varied across age groups. For instance, flavoured drinks contribute almost 50 % of the total intake for adolescents, whereas jam represents a higher contribution for adults and confectionery for children. A change in the major contributors of exposure from adolescents to adults was also observed. Further research is warranted on the relationship between the dietary intake of low/no-calorie sweeteners and specific food consumption patterns in specific subpopulations.

Despite the high steviol glycoside concentrations found in tabletop sweetener samples, the contribution of tabletop sweetener category to the total steviol glycosides exposure did not reach 2 % for the adult population, as mentioned before. Exposure from the food supplement category represents a small contribution to the total exposure, and the results indicate that the adult population is more exposed to steviol glucoside from food supplements than the children and adolescent population.

### Uncertainty assessment

The sources of uncertainty related to Tier 3 (T3) assessment are presented in Table 6^(42)^. The number of interview days (*n* 2) considered for the chronic exposure assessment is generally accepted, but might be low. Additionally, the consumption data were dated from 2014, whereas the study was conducted in 2020. Consequently, it may not adequately represent the actual consumption habits of participants. This might have a large impact on the uncertainties in an unknown direction. An additional source of uncertainty arises from the small number of analytical samples, coupled with certain food categories. For instance, seven breakfast cereal samples were purchased and analysed. Therefore, the mean concentration calculated and used for the T3 scenarios is probably a realistic estimate of the steviol glycoside concentration in commercially available products. However, the ‘Jam of fruit/vegetables homemade’ products from BNFCS2014 were linked to only one analysed sample. The high steviol glycoside content of this ingredient used to prepare homemade jams involves a high level of uncertainty in the gap with reality. In product categories with a limited number of samples in the market, the coupling tends to overestimate the exposure owing to the low availability of commercial products. The restrictions/exceptions ‘only energy-reduced’ and ‘with no added sugar’ were not applied during the matching, resulting in overestimation of the exposure. Brand loyalty and food supplement intake slightly increased intake estimates. However, this was counteracted in the T3max and T3maxfs scenarios. Overall, because of the conservative approach in coupling sample concentrations with BNFCS2014 and aggregation at the level of the consumption data, Tier 3 scenarios are expected to provide an overestimated intake.

**Table 6.** Qualitative evaluation of the influence of uncertainties on Tier 3 (T3) exposure estimates

Uncertainty	
Only 2 interview days to estimate chronic exposure	---/+++
2014 survey	--/++
Analytical errors	-/+
Uncertainty coupling sample results with BNFCS2014	-/+++
Restrictions ‘only energy-reduced’ and ‘with no added sugar’	++
Food supplements	-/
Brand loyalty	-

--- large, -- medium, - small underestimation; +++ large, ++ medium, +small overestimation; / neutral.

The results corroborate other findings in the literature: (i) the exposure estimates could exceed the ADI for steviol glycosides in unrefined assessments (e.g. household budget or Tier 2); (ii) the exposure estimates do not exceed the ADI in refined assessment; (iii) children are among the most exposed population, and soft drinks are an important contributor to the exposure^(24–26,43–45)^. However, one may not extrapolate the Belgian findings for steviol glycosides to other countries without considering the local consumption patterns, products and market share of food brands and products. For example, desserts in Italy are the most important contributors to intake, much lower than that of the Belgian population^(24)^. In Ireland, solid food products are the most important contributors to steviol glycoside exposure^(25)^. In South Korea, adolescents and adults are the most exposed population in a specific scenario^(46)^.

## Conclusion

In conclusion, the intake of steviol glycosides was below the ADI in all age groups in the general Belgian population. Even with more conservative refined approaches (T3max and T3maxfs), the estimated daily intake remained below 20 % of ADI. Flavoured drinks, flavoured fermented milk products, jams, jellies and marmalades contributed the most to the total intake. Despite the high concentrations (up to 94 g/kg) of steviol glycosides in tabletop sweeteners, their contribution to the total intake remains very low because of their sole use by the adult population and low level of consumption. The impact of food supplement use on the total intake was also considered minimal.

## Supplementary material

For supplementary material accompanying this paper visit https://doi.org/10.1017/jns.2023.13. click here to view supplementary material
