# Supplementary material for: Tiered dietary exposure assessment of steviol glycosides in the Belgian population
Source: J Nutr Sci. 2023 Feb 21;12:e28. doi: 10.1017/jns.2023.13 (PMC9947756; doi:10.1017/jns.2023.13)
Supplement: Supplementary file 1 [file S2048679023000137sup001.docx]

**Supplementary Table 1: Purchased samples distribution within EC food categories and corresponding mean and maximum steviol glycosides concentration in analysed food products (results expressed as mg_stevio eq._ /kg_product_)**

| FCS-category number | FCS category ^(a)^   - BNFCS2014 FoodEx2 Name | Number of samples | Samples mean cc.  (mg/kg) | Samples max cc.  (mg/kg) |
| --- | --- | --- | --- | --- |
| 1.4 | Flavoured fermented milk products including heat-treated products | Tot: 8 |  |  |
|  | - Probiotic milk-like drinks | 1 | 47.94 | 47.94 |
|  | - Soya drink | 1 | 26.20 | 26.20 |
|  | - Yoghurt, Greek style | 2 | 63.80 | 65.01 |
|  | - Yoghurt, cow milk, plain | 4 | 29.56 | 33.85 |
| 3 | Edible ices | Tot: 18 |  |  |
|  | - Frozen yoghurt | 2 | 41.26 | 46.97 |
|  | - Ice cream, milk-based | 15 | 55.04 | 73.86 |
|  | - Sorbet | 1 | 55.28 | 55.28 |
| 4.2.2 | Fruit and vegetables in vinegar, oil, or brine |  |  |  |
| 4.2.4.1 | Fruit and vegetable preparations excluding compote |  |  |  |
| 4.2.5.1 | Extra jam and extra jelly |  |  |  |
| 4.2.5.2 | Jam, jellies and marmalades and sweetened chestnut puree | Tot: 7 |  |  |
|  | - Jam of fruit or vegetables homemade / kit | 1 | 99.93 | 99.93 |
|  | - Jam of fruit or vegetables | 6 | 5.31 | 8.02 |
| 4.2.5.3 | Other similar fruit or vegetable spreads |  |  |  |
| 5.1 | Cocoa and Chocolate products | Tot: 15 |  |  |
|  | - Bitter chocolate | 6 | 64.42 | 80.69 |
|  | - Milk chocolate | 8 | 8.85 | 17.78 |
|  | - Truffle mass | 1 | 27.77 | 27.77 |
| 5.2 | Other confectionery | Tot: 12 |  |  |
|  | - Chocolate spread | 3 | 59.24 | 89.25 |
|  | - Hard candies | 2 | 752.95 | 1,370.00 |
|  | - Liquorice candies | 1 | 692.20 | 692.20 |
|  | - Soft candies and analogues | 4 | 187.40 | 326.80 |
|  | - Candies (breath-freshening) | 2 | 706.00 | 1,300.00 |
| 5.3 | Chewing gum |  |  |  |
| 5.4 | Decorations, coatings and fillings, except fruit-based fillings |  |  |  |
| 6.3 | Breakfast cereals | Tot: 7 |  |  |
|  | - Muesli plain | 7 | 27.59 | 34.70 |
| 7.2 | Fine bakery wares | Tot: 4 |  |  |
|  | - Fruit cake | 2 | 2.77 | 3.28 |
|  | - Biscuits type essoblaten-wafer paper | 1 | 66.24 | 66.24 |
|  | - Biscuits, chocolate | 1 | 2.55 | 2.55 |
| 9.2 | Processed fish and fisheries products |  |  |  |
| 11.4 | Tabletop Sweeteners SG^(b)^ | Tot: 21 |  |  |
|  | - Sweetener SG in crystal form | 6 | 733.43 | 980.30 |
|  | - Sweetener SG in powder form | 7 | 6,457.57 | 11,081.00 |
|  | - Sweetener SG in tablet form | 8 | 59,141.50 | 94,069.00 |
| 12.4 | Mustard |  |  |  |
| 12.5 | Soups and broths |  |  |  |
| 12.6 | Sauces | Tot: 2 |  |  |
|  | - Tomato ketchup and related sauces | 1 | 99.68 | 99.68 |
|  | - Mayonnaise sauce | 1 | 87.45 | 87.45 |
| 13.2 | Dietary foods for special medical purposes |  |  |  |
| 13.3 | Dietary foods for weight control diets | Tot: 12 |  |  |
|  | - Single meal replacement for weight reduction | 4 | 102.78 | 166.55 |
|  | - Protein and protein components for sports people | 8 | 25.53 | 126.77 |
| 14.1.3 | Fruit nectars |  |  |  |
| 14.1.4 | Flavoured drinks | Tot: 61 |  |  |
|  | - Fruit soft drink, orange | 6 | 24.97 | 36.95 |
|  | - Juice, apple | 20 | 46.84 | 80.00 |
|  | - Soft drink, flavoured, no fruit | 1 | 44.11 | 44.11 |
|  | - Soft drink, isotonic/sport | 3 | 57.13 | 62.72 |
|  | - Fruit soft drink, orange | 22 | 20.00 | 32.08 |
|  | - Soft drink, with fruit juice (fruit content below the minimum for nectars) | 9 | 30.04 | 47.97 |
| 14.1.5.2 | Other |  |  |  |
| 14.2.1 | Beer and malt beverages | Tot: 7 |  |  |
|  | - Lager beer | 7 | 16.95 | 29.56 |
| 14.2.8 | Other alcoholic drinks |  |  |  |
| 15.1 | Potato-, cereal-, flour- or starch-based snacks | Tot: 2 |  |  |
|  | - Potato crisps or sticks | 2 | 8.74 | 14.39 |
| 15.2 | Processed nuts |  |  |  |
| 16 | Desserts |  |  |  |
| 17.1 | Food supplements supplied in a solid form | Tot: 21 |  |  |
|  | - Fibre supplements | 1 | 1.87 | 1.87 |
|  | - Herbal formulations and plant extracts | 2 | 1.57 | 2.10 |
|  | - Mineral only supplements | 2 | 2.72 | 3.93 |
|  | - Mixed supplements/formulations (adults) | 4 | 2.96 | 6.99 |
|  | - Mixed supplements/formulations (children) | 1 | 2.45 | 2.45 |
|  | - Other common supplements | 3 | 0.84 | 2.23 |
|  | - Vitamin only supplements (adults) | 5 | 0.29 | 0.48 |
|  | - Vitamin only supplements (children) | 3 | 0.15 | 0.21 |
| 17.2 | Food supplements supplied in a liquid form | Tot: 7 |  |  |
|  | - Herbal formulations and plant extracts | 6 | 1.71 | 3.32 |
|  | - Mixed supplements/formulations | 1 | 1.51 | 1.51 |

^(a)^ FCS, food categorisation system (food nomenclature) presented in Annex II to Regulation (EC) No. 1333/2008.

^(b)^ SG: steviol glycosides.
